# Hyperspectral-Imaging-Based ECNN-1D for Accurate Origin Classification of Fragrant Pears

**DOI:** 10.3390/foods15091552

**Published:** 2026-04-30

**Authors:** Zhihao Liang, Xiaoyang Zhang, Fei Tan, Ruoyu Di, Jinbang Zhang, Wei Xu, Pan Gao, Li Zhang

**Affiliations:** 1 College of Information Science and Technology, Shihezi University, Shihezi 832003, China; liangzhihao@stu.shzu.edu.cn (Z.L.); m18317843602@163.com (X.Z.); tfnszbd@163.com (F.T.); diruoyu@stu.shzu.edu.cn (R.D.); zhangjinbang@stu.shzu.edu.cn (J.Z.); 2 Key Laboratory of Physiological and Quality Control of Specialty Fruits and Vegetables, College of Agriculture, Shihezi University, Shihezi 832003, China; xuwei0412@shzu.edu.cn

**Keywords:** fragrant pears, geographical origin identification, hyperspectral, Efficient Channel Attention

## Abstract

Geographical origin identification of fragrant pears is crucial for ensuring fruit quality, protecting regional brand value, and maintaining market order. However, pears from different origins often exhibit highly similar appearance and physicochemical properties, making rapid and nondestructive identification challenging for traditional methods. This study proposes a hyperspectral origin identification method based on an enhanced one-dimensional convolutional neural network (ECNN-1D) incorporating an Efficient Channel Attention (ECA) mechanism, using visible–near-infrared (Vis–NIR) and short-wave infrared (SWIR) spectral data. To address the technical challenges of highly similar spectra, redundant features, and complex information distribution, ECNN-1D enhances discriminative spectral feature representation, overcoming limitations of conventional machine learning and standard deep learning models in feature extraction and classification stability. Systematic comparisons with machine learning models (LDA, RF, KNN, SVM) and deep learning models (VGG-1D, ResNet-1D, CNN-1D) showed that while all models performed well on Vis–NIR spectra, ECNN-1D achieved the highest test accuracy of 98.94% and F1 score of 98.95% on the more challenging SWIR spectra, outperforming other approaches. These results indicate that ECNN-1D enables high-precision, nondestructive origin identification of fragrant pears, with potential cost advantages, providing a reliable technical solution for fruit traceability and quality supervision.

## 1. Introduction

Fragrant pear is one of the representative fruits in China, rich in carbohydrates, dietary fiber, various vitamins, and mineral elements such as potassium and calcium. It is characterized by its sweet taste, crisp texture, and abundant juice and is often referred to as the “king of fruits” [1]. In recent developments, the brand value of high-quality fragrant pear production regions has continuously increased; however, issues such as origin confusion and counterfeit labeling have become increasingly prominent, disrupting market order and affecting consumer interests. Korla fragrant pear, Aksu fragrant pear, and Hebei Hongxiangsu Pear exhibit highly similar appearances and fruit characteristics, while their internal physicochemical differences are minimal. These factors, combined with environmental variations and the limited availability of samples, render traditional manual identification and single-feature-based methods insufficient for rapid and accurate geographical origin discrimination [2]. To address this need, in previous studies, various chemical analysis methods requiring sample processing have been applied, such as determining the contents of sugars, organic acids, and mineral elements, as well as employing stable isotope ratio analysis (SIRA) for elements like carbon, hydrogen, and oxygen [3,4]. Gas chromatography–mass spectrometry (GC–MS) and liquid chromatography methods have also been used to detect volatile compounds or metabolites to differentiate samples from different production regions [5]. Although these methods can provide relatively accurate analytical results, they are time-consuming, operationally complex, and costly, which limits their applicability in large-scale, rapid, and real-time detection scenarios [6]. Consequently, there is a pressing need for efficient, nondestructive, and high-throughput technologies for the identification of fragrant pears’ geographical origins.

Hyperspectral imaging captures the spectral information of each pixel across continuous narrow bands, simultaneously reflecting both the surface features and internal chemical constituents of the fruit. In the Vis–NIR region, spectral variations primarily reflect the surface color, ripeness, and certain shallow internal chemical components of the fruit [7]. In contrast, the SWIR region is more sensitive to internal water content, sugars, and other chemical components, due to the vibrational characteristics of C–H, O–H, and N–H groups producing distinct absorption peaks. These absorption features directly reveal changes in internal chemical composition, providing critical information for quality assessment and origin discrimination. Studies have shown that Vis–NIR spectroscopy (397–1175 nm), combined with image processing techniques, can effectively distinguish the geographical origins of four traditional Chinese peach varieties [8]. In the dual identification task of apple varieties and geographical origins, researchers collected full-band hyperspectral data and combined K-nearest neighbor (KNN) and support vector machine with radial basis function kernel (SVM-RBF) models to achieve high-precision discrimination among four production regions and ten apple varieties, demonstrating the effectiveness of spectral fingerprints in complex classification scenarios [9]. Furthermore, Fourier transform near-infrared spectroscopy (FT-NIRS) has shown significant advantages in the geographical origin identification of pear fruits. For major Chinese geographical indication pear varieties such as Laiyang pear, Dangshan pear, and Korla fragrant pear, FT-NIRS successfully constructed robust origin discrimination models [10]. Notably, the selection of spectral bands plays a critical role in model performance. In the classification of durian maturity, short-wave near-infrared (SWNIR, 450–1000 nm) and long-wave near-infrared (LWNIR, 860–1750 nm) spectral ranges were evaluated. The results indicated that LWNIR achieved significantly better classification performance than SWNIR because it contained more chemical information related to dry matter and starch conversion [11]. However, traditional shallow machine learning models often struggle to fully capture the complex nonlinear relationships and high-dimensional patterns inherent in hyperspectral data, limiting their accuracy and generalization in fruit origin identification tasks.

In contrast, deep learning approaches such as one-dimensional convolutional neural networks (1D-CNNs) have demonstrated stronger capabilities in automatic feature extraction and noise resistance in fruit dry matter prediction tasks [12]. Gao et al. [13] conducted geographical origin classification experiments for dried *Elaeagnus angustifolia* fruits using near-infrared hyperspectral imaging combined with a convolutional neural network (CNN), achieving classification accuracies exceeding 90% on the prediction set and outperforming traditional chemometric methods. Cui et al. [14] applied a Bayesian-optimized multi-task residual fully convolutional network (MRes-FCN) to classify the geographical origins of Ningxia goji berries, using visible–near-infrared (Vis–NIR, 400–1000 nm) and near-infrared (NIR, 1000–1700 nm) hyperspectral data. The study combined a denoising autoencoder (DAE) for data augmentation with PCA and gray level co-occurrence matrix (GLCM) for texture feature extraction. The network achieved classification accuracies of 95.54% and 96.43% using the full spectrum and the optimal feature spectrum, respectively, demonstrating strong origin identification capability. Cai et al. [15] applied deep learning combined with hyperspectral imaging to identify the geographical origin of *Radix Paeoniae Alba*, and the constructed deep network significantly improved discrimination performance among samples from different regions, confirming the effectiveness of deep models in modeling high-dimensional spectral data. Jiang et al. [16] combined visible–near infrared hyperspectral imaging with a spectral–image feature fusion convolutional neural network (S-IFCNN) to identify the origin of goji berries. In that study, a combination of minimum transform and watershed algorithms was used to segment attached berries, enabling the extraction of spectral and image features. Li et al. [17] used a multimodal convolutional neural network with cross-attention mechanisms (MTCNN) to effectively integrate hyperspectral information with spatial image features. By jointly modeling spectral and image information, the model achieved a test set classification accuracy of 99.88%, significantly outperforming conventional methods.

Although hyperspectral imaging combined with deep learning has achieved promising results in the geographical origin identification of agricultural products such as goji berries and *Radix Paeoniae Alba*, several challenges remain in the case of fragrant pear origin identification. First, traditional shallow machine learning approaches rely heavily on manually extracted spectral or statistical features. Their representational capacity is significantly constrained when dealing with subtle chemical differences among fragrant pear production regions and the complex nonlinear relationships inherent in high-dimensional spectral data, resulting in limited discriminative accuracy. Second, although some deep learning models possess automatic feature extraction capabilities, they are prone to overfitting under conditions of limited sample size and regional variations in spectral distributions. Furthermore, such models may overlook critical spectral bands essential for origin discrimination, thereby reducing their generalization performance across different production regions. To address these challenges, an enhanced one-dimensional convolutional neural network (ECNN-1D) incorporating an Efficient Channel Attention mechanism was developed for hyperspectral imaging-based geographical origin identification of fragrant pears. The specific objectives of this study are as follows:

1. Fragrant pear samples were collected from the main production regions of Korla, Aksu, and Hebei Hongxiangsu Pear, covering different fruit sizes and harvesting environments, to construct a hyperspectral dataset that provides a comprehensive sample basis for origin identification research.
2. Principal component analysis (PCA) and uniform manifold approximation and projection (UMAP) were employed to perform dimensionality reduction and visualization of high-dimensional spectral data, highlighting key spectral band features, improving origin discrimination capability, and reducing the influence of environmental noise.
3. An enhanced spectral feature extraction network, ECNN-1D, incorporating an Efficient Channel Attention mechanism was proposed. This mechanism adaptively adjusts channel weights while preserving spectral integrity, thereby strengthening key spectral band features related to origin discrimination and improving model robustness against spectral distribution shifts among different production regions.
4. A systematic comparison of traditional machine learning models (Linear Discriminant Analysis, LDA; Random Forest, RF; K-Nearest Neighbors, KNN; Support Vector Machine, SVM) and deep learning models (One-Dimensional VGG Network, VGG-1D; One-Dimensional Residual Network, ResNet-1D; One-dimensional Convolutional Neural Network, CNN-1D) was conducted to evaluate the effectiveness and robustness of different approaches for origin identification.

## 2. Materials and Methods

**Figure 1:**
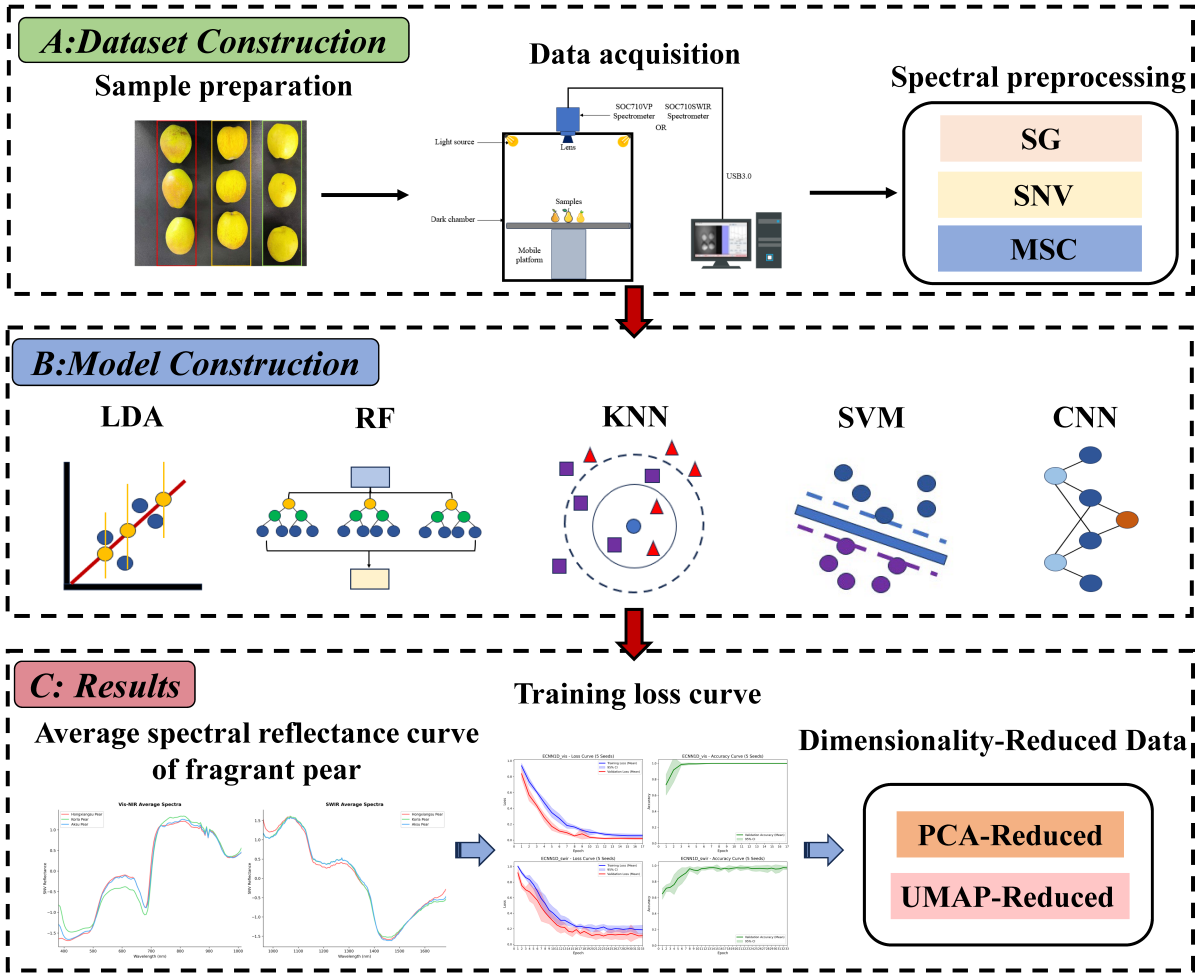
Overall workflow of this study.

This study mainly consists of three parts: hyperspectral dataset construction of fragrant pears, model development, and result analysis. The overall workflow is shown in Figure 1.

### 2.1. Sample Preparation

The experiment selected Korla fragrant pear and Aksu fragrant pear from Xinjiang and Hebei Hongxiangsu Pear as the research objects. These three types of pears were collected from standardized orchards located in Wensu County, Aksu Prefecture, Xinjiang (41.28° N, 80.25° E); Zhao County, Shijiazhuang City, Hebei Province (37.76° N, 114.78° E); and Yuli County, Korla City, Bayingolin Mongolian Autonomous Prefecture, Xinjiang (41.35° N, 86.27° E), respectively. Although both Korla and Aksu fragrant pears are produced in Xinjiang, notable differences in sunshine duration, diurnal temperature variation, and soil properties between the two regions result in subtle variations in their internal chemical composition and external characteristics. In contrast, Hongxiangsu Pears cultivated in Hebei differ significantly from the other two in terms of latitude, altitude, climate, and soil conditions, providing a more contrasting sample basis for investigating the effects of production regions on pear quality and for achieving rapid geographical origin identification.

**Figure 2:**
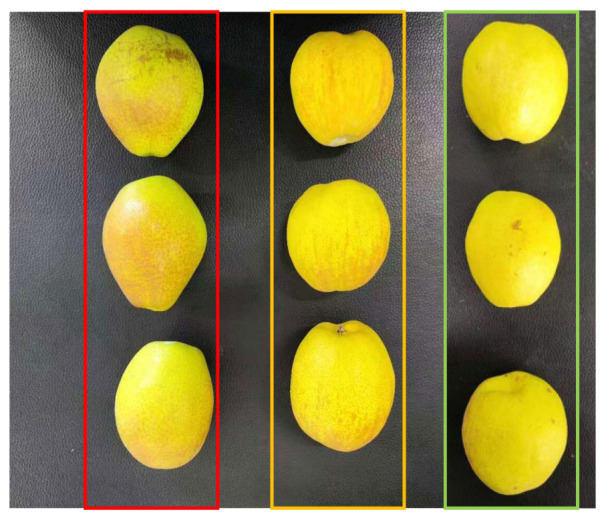
Fragrant pear samples: red box = Hebei Hongxiangsu Pear, yellow box = Aksu Pear (Xinjiang), green box = Korla Pear (Xinjiang).

All samples were harvested at natural maturity and immediately placed into specialized foam packaging boxes to minimize mechanical vibration or collision damage during transportation. To prevent quality deterioration caused by temperature fluctuations during transit, the fruits were transported to the laboratory by cold-chain air shipment under stable low-temperature conditions. Upon arrival, all samples were carefully inspected and manually screened, and fruits with visible defects, mechanical damage, or abnormal morphology were removed. This ensured that the samples used in subsequent experiments maintained high consistency in their initial physical condition, thereby reducing the influence of individual variability on experimental results. The selected samples were then cleaned using dust-free paper tissues to remove surface contaminants, and the fruit stems were uniformly removed to minimize interference from external impurities during hyperspectral reflectance data acquisition (Figure 2), thereby ensuring the stability and accuracy of the collected data.

To simulate practical storage conditions and examine quality changes of fragrant pears during different storage stages, the samples were promptly transferred to a professional constant-temperature storage facility at the College of Agriculture, Shihezi University (44.32° N, 86.07° E). The storage temperature was strictly maintained at 20 ± 0.5 °C, with relative humidity controlled between 60% and 65%, ensuring stable sample conditions without external environmental interference during storage. Table 1 presents the sample distribution of each pear variety across the four storage periods. For each variety, 60 samples were collected at each storage period, yielding a total of 720 samples, thereby ensuring a balanced dataset for subsequent hyperspectral analysis.

### 2.2. Hyperspectral Image Acquisition

**Figure 3:**
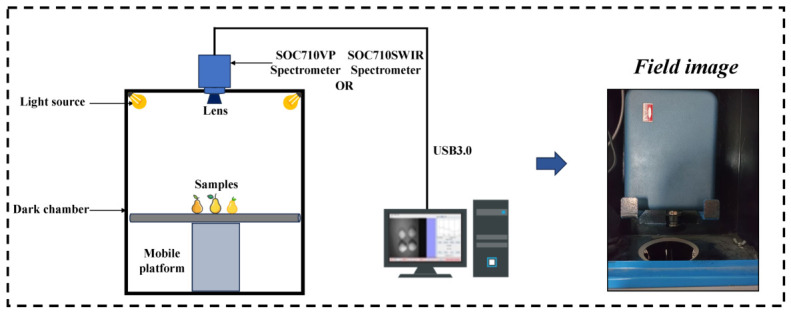
Hyperspectral imaging system.

Two hyperspectral imaging (HSI) systems were employed to acquire spectral images of pear samples in the Vis–NIR and SWIR spectral regions. The imaging system consisted of four components: an imaging module, an illumination module, a lifting platform module, and a software control module, as shown in Figure 3. During data acquisition, the integration time and camera gain were set to 10 ms and 1× for the Vis–NIR camera and 20 ms and 2× for the SWIR camera, respectively, to ensure optimal signal quality and prevent saturation. As samples were manually replaced between captures, no scanning speed parameter applied.

The imaging module utilized two hyperspectral cameras manufactured by Surface Optics Corporation (Surface Optics Corporation, San Diego, CA, USA), namely the SOC710-VP and SOC710-SWIR. Both cameras operate in a push-broom scanning mode and are equipped with sensors optimized for their respective spectral ranges, enabling multi-band image acquisition. The SOC710-VP camera covers a spectral range of 375–1045 nm with a spectral resolution of 4.09 nm and includes 128 spectral bands. It is equipped with a silicon-based CCD sensor. The SOC710-SWIR camera operates within a spectral range of 915–1700 nm with a spectral resolution of 2.72 nm and contains 288 spectral bands, using an InGaAs sensor. The detailed technical parameters of the hyperspectral cameras are listed in Table 2. To ensure imaging quality, an adjustable-height platform (Kaiser, model 5513RS10, Buchen, Germany) was used to precisely adjust the position and orientation of the samples, ensuring that samples of different sizes and shapes remained within the optimal field of view of the camera.

The illumination system consisted of four 75 W halogen reflector lamps (OSRAM, model MR11, Munich, Germany), which provide continuous spectral radiation in the range of 320–2500 nm, thereby covering the operating spectral range of the hyperspectral imaging system. This configuration ensured stable and uniform illumination conditions and reduced noise caused by uneven lighting. Spectral calibration was performed using a polytetrafluoroethylene (PTFE) diffuse reflectance reference panel with a reflectance of 18%. The reference panel exhibits good Lambertian reflectance characteristics and maintains stable reflectance across the entire spectral range of the hyperspectral imaging system, making it a reliable radiometric calibration standard. Calibration using the reference panel effectively reduced the influence of environmental light variation and instrument background noise on the measurements.

Hyperspectral image acquisition and preliminary processing were conducted using HyperScanner acquisition software (HyperScanner 2.0) and SRAnalysis analysis software (SRAnal710e, version 3.0) provided by SOC. Subsequently, ENVI 5.1 software (ITT Visual Information Solutions, Boulder, CO, USA) was used to extract the region of interest (ROI) from each hyperspectral image to remove irrelevant background information. To ensure temporal coverage during storage, spectral data were collected at four-day intervals. As shown in Table 1, at each sampling time, 60 samples were randomly selected from each pear variety, resulting in a total of 720 samples (3 varieties × 4 storage stages × 60 samples). After excluding samples with defects or abnormal spectral responses, 660 valid samples were retained for subsequent analysis. To objectively evaluate the model, the dataset was split at the spectral level rather than the fruit level into training, validation, and test sets at a 6:2:2 ratio, using stratified sampling to maintain class balance. As multiple spectra were collected from each fruit, spectra from the same fruit may be distributed across different subsets. This spectral-level splitting allows full utilization of the data and enables performance evaluation at the spectral level.

### 2.3. Spectral Preprocessing

To further improve the quality of the spectral data and minimize the effects of instrumental noise, background interference, and stray light on model development, multiple preprocessing techniques were applied to the raw spectra, including Savitzky–Golay (SG) smoothing [18], standard normal variate (SNV) transformation [19], and multiplicative scatter correction (MSC) [20]. In the model development stage, these methods were applied in combination, forming a joint preprocessing pipeline that effectively suppresses noise and interference, improves the quality of spectral data, and ensures the accuracy and robustness of model training.

SG smoothing was employed to suppress random noise in the spectra by fitting a local polynomial within a moving window. In this study, a third-order polynomial with a window size of 5 was used, effectively reducing high-frequency noise while preserving subtle spectral features associated with the chemical composition and geographical origin of the pears. SNV was applied to normalize each sample spectrum, mitigating intensity variations caused by particle scattering. This method standardizes the spectral data based on the mean and standard deviation of each spectrum, ensuring that all wavelengths follow a standard normal distribution, thereby eliminating intensity offsets arising from differences in peel thickness or surface texture. This enhances the comparability of spectra across samples and improves the accuracy and robustness of subsequent quality detection models. MSC was utilized to correct spectral shifts and intensity variations induced by surface scattering effects. By performing linear regression of each sample spectrum against a reference spectrum, additive and multiplicative scatter components were estimated and corrected. This procedure effectively reduces non-chemical variations due to scattering, allowing the corrected spectra to more accurately reflect the chemical and physical properties of the samples. MSC preprocessing is particularly critical for geographical origin identification and quality assessment, as it emphasizes subtle chemical differences among samples.

### 2.4. Spectral Feature Extraction Methods

#### 2.4.1. Principal Component Analysis

PCA is a widely used multivariate statistical method for dimensionality reduction and feature extraction in high-dimensional data [21], transforming correlated variables into a set of orthogonal principal components (PCs) where the first few components retain most of the variance information; in this study, PCA was applied to hyperspectral data from the Vis–NIR and SWIR ranges to reduce redundancy and noise prior to feature visualization and model training, projecting the data into a lower-dimensional space while retaining principal components explaining the majority of variance, thereby reducing computational complexity, improving training efficiency, and preserving essential spectral information.

#### 2.4.2. Uniform Manifold Approximation and Projection

UMAP is a nonlinear dimensionality reduction method based on manifold learning, designed to map high-dimensional data into a low-dimensional space for visualization and feature compression [22]. UMAP effectively preserves both global data structure and local neighborhood relationships and offers higher computational efficiency and scalability compared with t-distributed Stochastic Neighbor Embedding (t-SNE). The method constructs a graph reflecting the connectivity of the data manifold and optimizes its low-dimensional embedding via stochastic gradient descent, preserving local and global structures simultaneously. UMAP has been shown to provide competitive visualization quality with superior runtime performance and scalability compared with other nonlinear dimensionality reduction methods, making it suitable for large high-dimensional datasets and providing informative low-dimensional representations for visualization, classification, and clustering analyses [23]. In this study, UMAP was combined with PCA to explore the latent structures and class distributions in the hyperspectral data of Korla, Aksu, and Hongxiangsu Pears, providing a robust low-dimensional representation for subsequent deep feature extraction.

### 2.5. Traditional Machine Learning Models

To evaluate the performance of conventional approaches for fragrant pear geographical origin identification, four widely used traditional machine learning models were selected in this study: LDA [24], RF [25], KNN [26], and SVM [27].

LDA is a classical linear classification method that maximizes the ratio of between-class variance to within-class variance to achieve linear projection and class separation, making it suitable for high-dimensional but linearly separable data. RF is a nonlinear ensemble method based on decision trees, which constructs multiple trees and aggregates their predictions via majority voting, effectively capturing nonlinear relationships, reducing overfitting, and providing inherent feature selection capability. KNN is a non-parametric, instance-based classifier that assigns labels to unknown samples based on proximity to neighboring samples, offering intuitive interpretation, though its performance is sensitive to data dimensionality and sample distribution. SVM, grounded in the structural risk minimization principle, seeks the optimal hyperplane to maximize class margins, making it particularly effective for small-sample, high-dimensional, and nonlinearly separable problems. In this study, these traditional machine learning methods were applied to hyperspectral data of fragrant pears to evaluate the performance of different models in pear geographical origin identification and to provide a reference for subsequent comparison with deep learning approaches.

### 2.6. Deep Learning Models

In this study, several representative deep learning architectures were employed to extract spectral features and classify the geographical origins of fragrant pears. These models exhibit distinct feature extraction strategies and network structures, allowing spectral data to be processed from different perspectives and providing a comparative basis for evaluating the advantages of the proposed ECNN-1D model in feature representation and classification performance.

#### 2.6.1. One-Dimensional Residual Network

In this study, a lightweight ResNet-1D was developed to extract spectral features and classify the geographical origins of fragrant pears [28]. The model operates directly on spectral sequences represented as tensors of shape (batch_size, 1, *L*), where batch_size denotes the number of samples per input batch, 1 represents the single channel of the spectral signal, and *L* indicates the number of spectral bands. The network backbone consists of cascaded residual blocks, each containing two 1D convolutional layers with batch normalization and ReLU activation, while residual connections facilitate gradient propagation and feature fusion. For blocks where the input and output channels differ, a 1×1 convolution is applied to align dimensions. The extracted features are aggregated via global average pooling into fixed-length vectors, which are subsequently fed into a fully connected layer. Finally, a Softmax function produces the predicted probabilities for the three fragrant pear origins, enabling accurate and robust classification.

#### 2.6.2. One-Dimensional VGG Network

**Figure 4:**
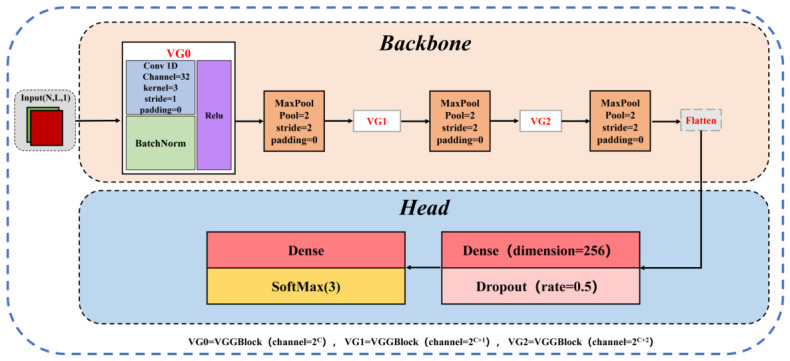
VGG -1D Network Architecture.

Based on the classical VGG network [29], a one-dimensional convolutional neural network for spectral classification, VGG-1D, was developed by adapting the convolution operations and input representation to the sequential nature of one-dimensional spectral data, as illustrated in Figure 4.

Unlike the original VGG, which employs two-dimensional convolutions for image data, VGG-1D applies one-dimensional convolution layers directly to spectral band sequences, with each sample represented as a three-dimensional tensor (n,l,1), where *n* denotes the number of samples, *l* the spectral band length, and the single channel corresponds to the one-dimensional spectral signal. This representation effectively captures local correlations along the wavelength dimension. The network retains the VGG “convolution–pooling” stacking paradigm, consisting of three convolutional blocks. Each block contains two Conv1D layers, batch normalization, and a max-pooling layer. Convolutional kernels are uniformly set to size 3, and ReLU activation functions are used to enhance nonlinear feature representation. Max-pooling layers progressively reduce feature length, lower computational complexity, and suppress noise. To mitigate overfitting and improve generalization, dropout regularization is applied after each convolutional block and fully connected layer. After multi-layer convolutional feature extraction, the feature maps are flattened and fed into fully connected layers, with a final Softmax function generating the predicted probabilities for the three fragrant pear geographical origins.

#### 2.6.3. Enhanced One-Dimensional Convolutional Neural Network

To further enhance the spectral discriminative capability among the three fragrant pear varieties, this study incorporated the Efficient Channel Attention (ECA) mechanism [30] into a CNN-1D [31], resulting in a spectral feature enhancement model termed ECNN-1D. The model adaptively emphasizes the importance of different spectral channels by modeling local dependencies along the channel dimension, thereby strengthening key spectral bands that are most informative for fragrant pear geographical origin classification.

**Figure 5:**
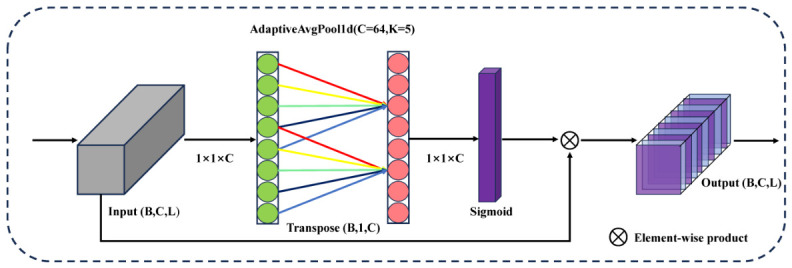
ECA Attention Module.

Unlike conventional channel attention methods, the ECA mechanism avoids explicit dimensionality reduction, enabling efficient cross-channel interaction while preserving the integrity of spectral features. It is lightweight, structurally simple, and particularly suitable for high-dimensional, information-dense one-dimensional spectral data. The architecture of the ECA module is illustrated in Figure 5. The input spectral feature tensor has the shape (B,C,L), where *B* denotes the batch size, *C* the number of channels, and *L* the spectral band length. The ECA module first applies adaptive average pooling (AdaptiveAvgPool1d) along the spectral dimension to obtain a global channel descriptor. The resulting feature is then reshaped to (B,1,C) to match the input requirements of the one-dimensional convolution along the channel dimension. Subsequently, a one-dimensional convolution with kernel size *k* models local dependencies between adjacent channels. The kernel size *k* is adaptively determined based on the number of channels *C* according to the following formula:

(1) $$k=\psi \left(C\right)=\left\lfloor \frac{\left|\log_{2}\left(C\right)+1\right|}{2}\right\rfloor$$

The kernel size is further constrained to be an odd number to ensure the symmetry of the convolution operation, thereby avoiding uncertainties associated with manually setting hyperparameters. In the experimental setup of this study, when the number of channels C=16 or 32, the adaptively determined kernel size is k=3; when C=64, the kernel size is k=5. The convolution output is then passed through a Sigmoid activation function to generate the channel-wise weight vector, which is multiplied with the original input features along each channel. This produces the enhanced spectral features while retaining the original tensor shape. This process effectively emphasizes spectral channels with high discriminative value for pear variety classification while suppressing redundant or noisy bands.

**Figure 6:**
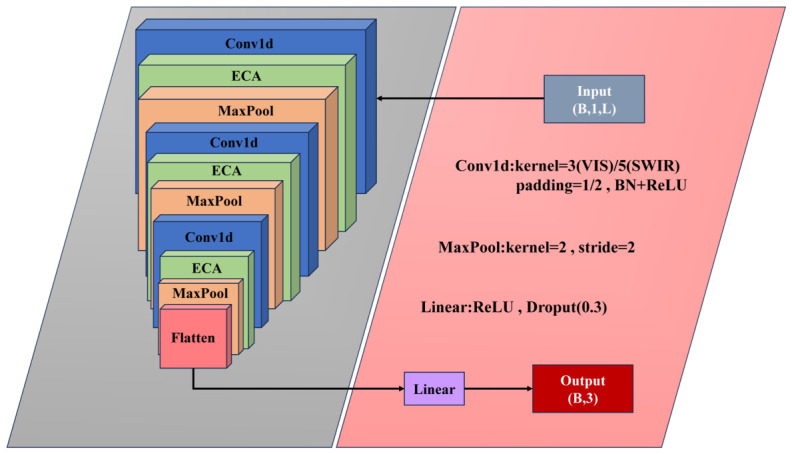
ECNN -1D Network Structure.

At the network architecture level, ECNN-1D is composed of multiple cascaded one-dimensional convolutional blocks, each sequentially comprising a Conv1D layer, batch normalization, ReLU activation, the ECA attention module, and max pooling, enabling hierarchical extraction and compression of spectral features, as shown in Figure 6. In the shallow layers, a smaller number of convolutional channels is used to capture the fundamental reflective characteristics of fragrant pear spectra; as the network depth increases, the number of channels is progressively expanded to enhance the representation of higher-level discriminative features and nonlinear spectral variations. Finally, adaptive average pooling compresses the high-dimensional feature maps into fixed-length vectors, which are fed into a fully connected classifier to output predicted probabilities for each fragrant pear variety, achieving accurate classification and traceability of fragrant pears’ geographical origins.

### 2.7. Model Evaluation Metrics

For the multi-class classification task of fragrant pear geographic origin identification, classification accuracy (Accuracy) was adopted as the primary evaluation metric, defined in Equation (2). Accuracy is calculated as the ratio of correctly classified samples to the total number of samples, providing an overall measure of the model’s ability to correctly identify pears from different origins. Higher accuracy indicates stronger overall discriminative performance and predictive stability in the fragrant pear origin classification task.

(2) $$\mathrm{Accuracy}=\frac{N_{\mathrm{correct}}}{N_{\mathrm{total}}}$$

Here, $N_{\mathrm{correct}}$ represents the number of correctly classified samples, and $N_{\mathrm{total}}$ denotes the total number of samples in the test set. However, for multi-class classification problems—particularly when the spectral differences among pears from different origins are subtle—relying solely on accuracy may not fully reflect the model’s discriminative capability across all classes. Therefore, precision, recall, and F1-score are further introduced as complementary evaluation metrics to assess classification performance from multiple perspectives. For the *i*-th class, precision, recall, and F1-score are calculated as follows:

(3) $${\mathrm{Precision}}_{i}=\frac{TP_{i}}{TP_{i}+FP_{i}},{\mathrm{Recall}}_{i}=\frac{TP_{i}}{TP_{i}+FN_{i}}$$

(4) $$F1{\text{-score}}_{i}=\frac{2\times {\mathrm{Precision}}_{i}\times {\mathrm{Recall}}_{i}}{{\mathrm{Precision}}_{i}+{\mathrm{Recall}}_{i}}$$

Here, $TP_{i}$, $FP_{i}$, and $FN_{i}$ represent the number of true positives correctly classified for class *i*, the number of false positives incorrectly predicted as class *i*, and the number of false negatives misclassified into other classes, respectively. Precision reflects the reliability of the model’s predictions for samples from a given fragrant pear origin, recall measures the model’s ability to detect samples from that origin, and the F1-score balances the two, providing a more comprehensive evaluation of the model’s classification performance in the fragrant pear geographical origin identification task.

### 2.8. Experimental Setup

All data analysis and numerical computations in this study were performed using Python 3.12. Traditional machine learning models were implemented with scikit-learn 1.8.0, while deep learning models were constructed using PyTorch 2.5.1. To reduce the influence of random data splits, each experiment was repeated under five different random seeds, and the final results are reported as the mean ± standard deviation (mean ± std) of these five runs.

During model training, the AdamW optimizer was employed for parameter updates, with weight decay applied to mitigate overfitting. An early stopping mechanism was also introduced to improve training efficiency and prevent excessive overfitting: training was halted if the validation accuracy did not improve over 10 consecutive epochs, and the model parameters corresponding to the best validation performance were saved for final testing. The batch size, initial learning rate, weight decay, and other training hyperparameters are summarized in Table 3. All experiments were conducted on a workstation equipped with an Intel Core i7-9700K CPU (3.60 GHz, Intel Corporation, Santa Clara, CA, USA), 32 GB of RAM, and an NVIDIA GeForce GTX 1080 Ti GPU (NVIDIA Corporation, Santa Clara, CA, USA).

## 3. Results

### 3.1. Sample Spectral Analysis

**Figure 7:**
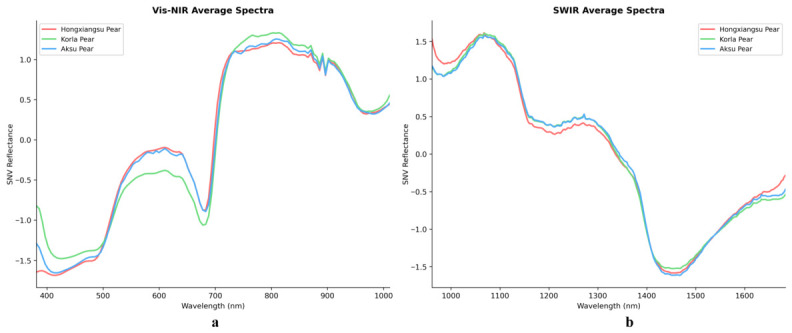
Mean spectral curves of fragrant pears from three origins. (**a**) Vis-NIR average spectra; (**b**) SWIR average spectra.

Spectral data of the three fragrant pear varieties were extracted from the Vis–NIR (376–1073 nm) and SWIR (915–1699 nm) hyperspectral images. Because the raw spectra exhibited significant noise at both ends, these bands were removed, retaining only the central effective bands, which were then processed using SNV transformation. The resulting mean spectra for Vis–NIR and SWIR are shown in Figure 7.

For the Vis–NIR spectra, after noise removal, the retained wavelength range was 381–1016 nm, comprising 123 bands; for the SWIR spectra, the retained range was 959–1684 nm, comprising 267 bands. As shown in Figure 7a, the spectral reflectance of Hongxiangsu Pear in the 400–500 nm region was clearly lower than that of Aksu Pear and Korla Pear, reflecting differences in chlorophyll and carotenoid content among the varieties [32]. All three pear varieties exhibited similar peaks around 600 nm and a red-edge absorption valley near 700 nm, which may be influenced by fruit peel cellular structure. Subtle differences between Aksu Pear and Korla Pear appeared in the 580–670 nm range, reflecting minor variations in flavonoid and soluble sugar content. In the 700–1000 nm near-infrared region, the spectra of Hongxiangsu Pear largely overlapped with those of Aksu Pear, showing only slight differences from Korla Pear, mainly manifested as minor variations in near-infrared absorption intensity. Figure 7b presents the mean spectral features of the SWIR region: a prominent reflection peak appeared near 1100 nm, and an absorption valley occurred around 1450 nm, corresponding to overall spectral responses of carbohydrates, lipids (C–H bond vibrations), and water content [33]. The spectra of Aksu Pear and Korla Pear almost completely overlapped from 1160–1350 nm, with only minor differences emerging beyond 1400 nm, possibly due to the similarity in their cultivation environments. Overall, the Vis–NIR and SWIR spectral curves alone do not allow intuitive discrimination among the three pear origins, and further spectral analysis combined with modeling approaches is required for accurate classification.

### 3.2. Exploratory Analysis of Spectral Data

**Figure 8:**
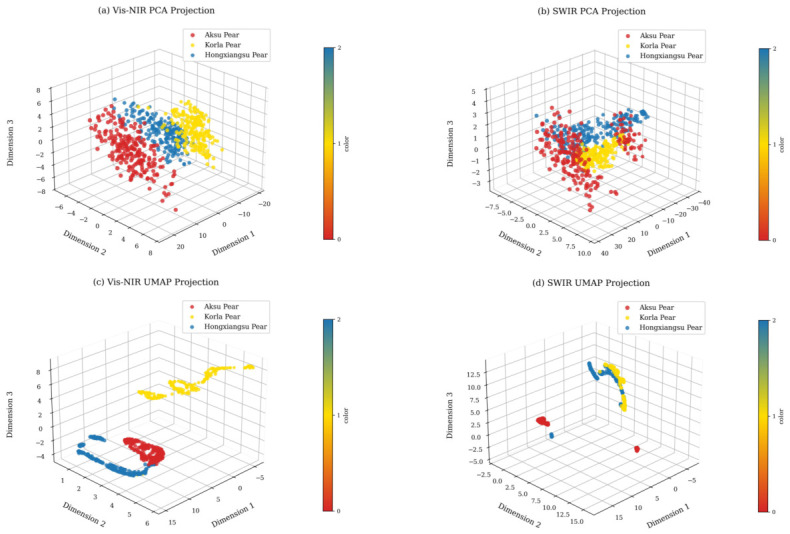
Three-dimensional projections of spectral data from fragrant pears of different origins under PCA and UMAP dimensionality reduction.

In this study, PCA was separately applied to the Vis–NIR and SWIR spectral data. For the Vis–NIR data, the first three principal components accounted for 80.9%, 10.7%, and 4.1% of the variance, with a cumulative contribution of 95.7%; for the SWIR data, the first three components contributed 74.9%, 9.7%, and 6.1%, with a cumulative contribution of 90.7%, indicating that the first three components effectively capture the majority of spectral information. As shown in Figure 8a, in the Vis–NIR spectral space, Korla Pear and Aksu Pear samples form relatively distinct clusters, whereas Hongxiangsu Pear partially overlaps with these two groups. Figure 8b shows that in the SWIR spectral space, a similar clustering trend is observed among the three fragrant pear varieties, but the overlap between Aksu Pear and Korla Pear increases, and some boundary samples of Hongxiangsu Pear intersect with others. Overall, the distributions of different-origin fragrant pear samples in single spectral spaces still exhibit notable overlap, which may affect subsequent origin classification accuracy. A similar phenomenon has been reported in hyperspectral studies on peach geographical origin discrimination, where even after PCA for dimensionality reduction, some samples may still overlap, thereby affecting classification performance [8].

UMAP was further employed to reduce the spectral data to three dimensions for comparative visualization. For the Vis–NIR projection (Figure 8c), the reduced data form clear clustering structures: Korla Pear, Aksu Pear, and Hongxiangsu Pear each cluster tightly with distinct intergroup boundaries, and only minor local overlaps are observed. Notably, Korla Pear is relatively distant from the other two groups, forming a relatively independent distribution in three-dimensional space, which facilitates differentiation to some extent. In contrast, the UMAP projection based on SWIR data (Figure 8d) shows more dispersed data points, with indistinct clustering structures and increased inter-sample overlap, resulting in poor overall clustering and making it difficult to clearly discriminate between fragrant pear origins. This phenomenon is consistent with previous studies, as the Vis–NIR spectral range is more sensitive to surface chemical components and pigments, and therefore generally exhibits stronger discriminative capability than the SWIR range in agricultural product classification tasks [34].

### 3.3. Classification Results Based on Full-Spectrum Data

Table 4 presents the performance of fragrant pear origin classification models based on full-band Vis–NIR and SWIR hyperspectral data. Overall, all models achieved high classification accuracy, indicating that the full-band hyperspectral data contain rich and discriminative features capable of effectively supporting the identification of fragrant pears from different origins.

For the Vis–NIR spectral data, most models achieved near-perfect classification results. Specifically, LDA, SVM, VGG-1D, and ECNN-1D attained 100.00% accuracy on both the validation and test sets, with corresponding P, R, and F1 also at 100.00%. RF and KNN achieved test set accuracies of 99.85% and 99.55%, respectively, while ResNet-1D and CNN-1D achieved 99.55% and 99.70%, with P, R, and F1 all exceeding 99.5%. These results demonstrate that Vis–NIR spectra possess strong discriminative capability for origin classification, likely due to the sensitivity of this spectral range to surface pigments and certain shallow chemical components of the fruit. This observation is consistent with previous studies on agronomic crops, including maize, sugarcane, coffee, rapeseed, wheat, and tobacco, in which Vis–NIR spectroscopy exhibited high sensitivity to peel pigments and to shallow water- and sugar-related absorption features, thereby enabling superior classification performance [35]. For SWIR spectral data, model performance was slightly lower than that of Vis–NIR, but all evaluation metrics remained above 95%. This is consistent with prior research showing that the SWIR range is more sensitive to deep chemical components such as starch, cellulose, and organic acids [36], but variations in signal-to-noise ratio and sample scattering may reduce model stability. Among traditional machine learning models, LDA achieved the highest test set accuracy of 97.73%, with corresponding P, R, and F1 scores of 97.74%, 97.78%, and 97.73%, respectively. Among deep learning models, VGG-1D, ResNet-1D, and CNN-1D achieved test set accuracies of 97.88%, 95.91%, and 98.03%, respectively. ECNN-1D performed best, achieving 99.09% and 98.94% accuracy on the validation and test sets, with P, R, and F1 all approaching 99%.

In summary, Vis–NIR spectra slightly outperform SWIR spectra in fragrant pear origin classification. Moreover, deep learning models exhibit superior capability in modeling high-dimensional spectral features. By incorporating a channel attention mechanism, ECNN-1D can adaptively enhance spectrally discriminative channels, resulting in stable and outstanding classification performance across both spectral types.

### 3.4. Ablation Study of the ECA Module

To further validate the methodological contribution of the proposed ECNN-1D model, the ECA module was incorporated into three representative deep learning architectures, including VGG-1D, ResNet-1D, and CNN-1D, to conduct ablation experiments. The quantitative results are presented in Table 5.

For the Vis–NIR dataset, all models achieved near-perfect classification performance, indicating that the spectral separability among different classes in this wavelength range is relatively high. Nevertheless, after introducing the ECA module, some models still exhibited performance improvements. Specifically, ResNet-1D achieved a testing accuracy of 99.55%, while ResNet-1D+ECA maintained a comparable performance with a testing accuracy of 99.39%, demonstrating stable classification capability. Meanwhile, CNN-1D improved from 99.70% to 100.00% after integrating the ECA module, indicating that the ECA mechanism can enhance the extraction of discriminative features even when spectral separability is high. Ultimately, ECNN-1D achieved 100.00% across all evaluation metrics, demonstrating excellent stability and robustness. More pronounced improvements were observed in the more challenging SWIR dataset. Specifically, the testing accuracy of VGG-1D increased from 97.88% to 98.33% after incorporating the ECA module. Similarly, CNN-1D improved from 98.03% to 98.94% after introducing the ECA module, further confirming the effectiveness of channel attention in capturing informative spectral features. Although ResNet-1D+ECA showed slightly lower testing accuracy compared to the baseline model, its validation accuracy improved from 97.42% to 98.79%, suggesting that the ECA module enhances feature representation, while performance variation may be influenced by architectural characteristics and data distribution. Overall, ECNN-1D achieved the highest classification accuracy of 98.94% on the SWIR dataset, outperforming all comparative models.

### 3.5. Model Performance Analysis

**Figure 9:**
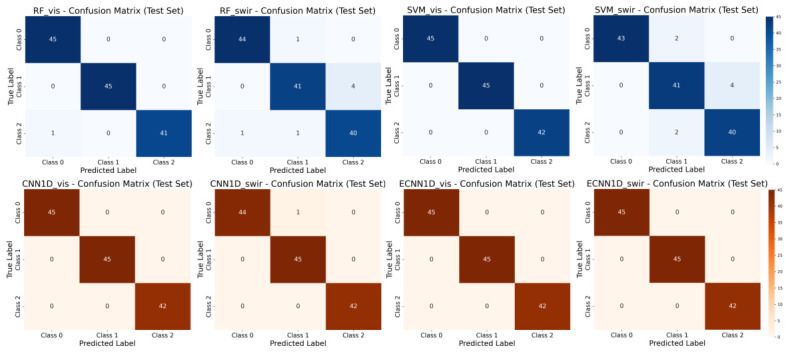
Confusion matrices of representative models on Vis–NIR and SWIR spectral datasets. Class 0: “Aksu Pear”; Class 1: “Korla Pear”; Class 2: “Hongxiangsu Pear”.

To more intuitively illustrate the model performance on the test set, Figure 9 presents the confusion matrices of representative models (RF, SVM, CNN-1D, and ECNN-1D) on the Vis–NIR and SWIR spectral datasets. For the Vis–NIR spectra, all models achieved high classification accuracy. In the confusion matrices of ECNN-1D, CNN-1D, and SVM, the diagonal elements almost completely corresponded to correctly classified samples, while only the RF model exhibited a small number of misclassifications, mainly where “Hongxiangsu Pear” was misclassified as “Aksu Pear,” although the overall classification boundaries remained clear. For the SWIR spectra, classification confusion slightly increased. The RF and SVM models showed minor misclassifications, including “Korla fragrant pear” being misclassified as “Hongxiangsu Pear” and “Aksu Pear” being misclassified as “Korla fragrant pear.” These misclassification phenomena may be related to the similarity in environmental conditions and varietal origins across different production regions. Korla and Aksu are both located in Xinjiang, China, where abundant sunlight, large diurnal temperature variation, and arid climate conditions favor sugar accumulation in fruits, resulting in similarities in physicochemical composition between the two types of pears. Meanwhile, the SWIR band is sensitive to internal components such as water, sugars, and organic compounds. Since Korla fragrant pears and Aksu Pears exhibit similarities in these components, a certain degree of spectral overlap occurs in the SWIR band, leading to minor misclassifications. In addition, Hongxiangsu Pear is a hybrid cultivar derived from Korla fragrant pear and Goose pear. Because its genetic background contains genes from Korla fragrant pear, similarities in internal physicochemical composition and spectral characteristics may exist, which could also contribute to slight confusion. In contrast, the CNN-1D and ECNN-1D models maintained higher stability with only a few misclassified samples, indicating that deep learning models can extract more discriminative nonlinear spectral features and exhibit stronger robustness when handling spectral overlap caused by environmental conditions and genetic similarities among varieties.

**Figure 10:**
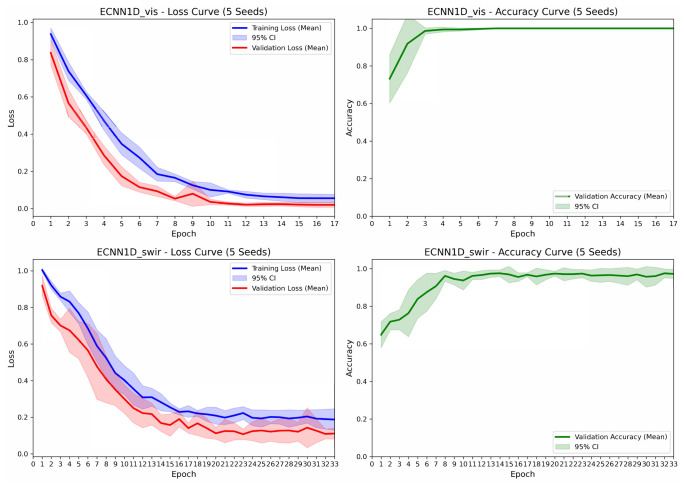
Training curves of ECNN-1D on Vis–NIR and SWIR spectral data (averaged over 5 random seeds, 95% confidence interval).

Taking the best-performing ECNN-1D as an example, Figure 10 shows its training curves on Vis–NIR and SWIR spectra. During Vis–NIR training, both training and validation losses decreased rapidly with increasing epochs and stabilized around the 10th epoch; validation accuracy quickly rose to near 100% in the early stages, with very narrow 95% confidence intervals, indicating stable convergence without noticeable overfitting. During SWIR training, the training and validation losses also showed a continuous decline, with validation accuracy gradually reaching approximately 99%. Although convergence was slightly slower than for Vis–NIR, the 95% confidence intervals remained narrow, indicating high consistency across different random seeds and demonstrating strong generalization ability and stability of the model.

### 3.6. Classification Results Based on Dimensionality-Reduced Data

#### 3.6.1. Principal Component Analysis-Based Feature Modeling

Although high classification accuracy for fragrant pear origin identification can be achieved using full-spectrum hyperspectral data, such data still contain substantial redundant information. To reduce data dimensionality and improve model efficiency, PCA was applied to the raw spectral data, retaining the first 20 principal components for subsequent modeling, and the results are summarized in Table 6.

**Figure 11:**
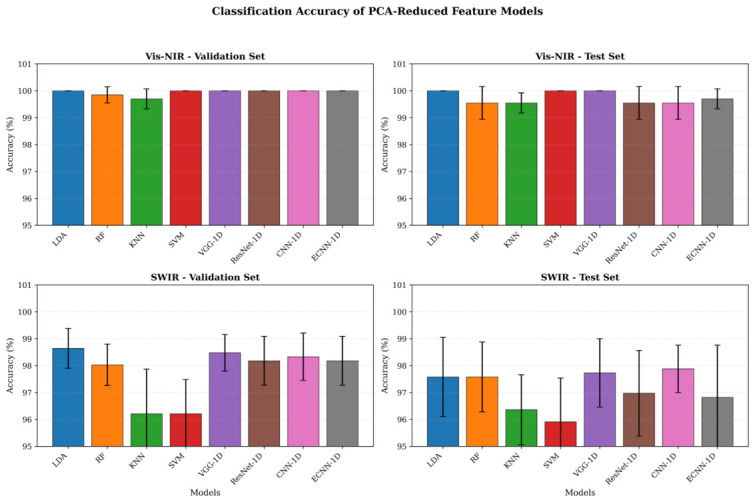
Comparison of classification accuracy for fragrant pear origin identification models based on PCA-reduced features.

For Vis–NIR spectra, all models exhibited very high classification performance, with validation set accuracy approaching 100% and test set accuracy ranging from 99.55% to 100.00%. Among them, ECNN-1D achieved 100.00±0.00% on the validation set and 99.70±0.37% on the test set, demonstrating stable and excellent classification capability. For SWIR spectra, overall performance of the models was slightly lower than that of Vis–NIR, yet high recognition accuracy was maintained, with test accuracies primarily between 95.91% and 97.88%. In this case, CNN-1D performed best, with a test accuracy of 97.88±0.88%, while ECNN-1D achieved 96.82±1.94%. Overall, PCA-reduced features allowed all models to achieve high classification performance on both spectral types, with Vis–NIR spectra yielding superior identification results compared to SWIR. Moreover, deep learning models consistently outperformed traditional machine learning approaches, further validating the effectiveness of the proposed models for fragrant pear origin identification. The comparative performance of different models is presented in Figure 11.

#### 3.6.2. Uniform Manifold Approximation and Projection-Based Feature Modeling

To further explore the application of dimensionality reduction in fragrant pear origin identification, this study employed UMAP to reduce the spectral data to 20 dimensions, which were then used as input features for both traditional machine learning and deep learning models. The experimental results are presented in Table 7.

**Figure 12:**
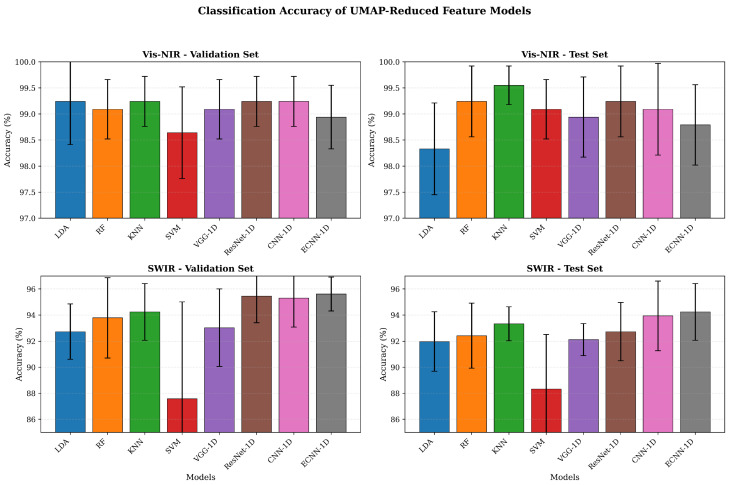
Comparison of classification accuracy for fragrant pear origin identification models based on UMAP-reduced features.

For Vis–NIR spectra, all models achieved high classification accuracy, with validation set accuracies ranging from 98.64% to 99.24% and test set accuracies between 98.33% and 99.55%. Among them, KNN achieved the highest test accuracy of 99.55 ± 0.37%, while RF and ResNet-1D also reached 99.24 ± 0.68%, indicating that UMAP-reduced Vis–NIR features effectively preserved the origin-specific spectral differences of the pears. In contrast, models based on SWIR spectra exhibited slightly lower classification performance, with test set accuracies mainly distributed between 88.33% and 94.24%. Among these, ECNN-1D performed best, achieving a test accuracy of 94.24 ± 2.17%, followed by CNN-1D (93.94 ± 2.67%) and KNN (93.33 ± 1.30%), whereas SVM achieved the lowest test accuracy of 88.33 ± 4.19%. Overall, models constructed with UMAP-reduced features achieved satisfactory fragrant pear origin identification for both spectral types, with Vis–NIR spectra outperforming SWIR spectra. Deep learning models demonstrated relatively stable performance on SWIR data, with ECNN-1D achieving the highest recognition accuracy, further confirming the effectiveness of the proposed approach for fragrant pear origin classification. The comparative performance of different models is presented in Figure 12.

## 4. Discussion

### 4.1. Overview of Key Findings and Novel Contributions

In this study, we proposed and systematically evaluated the one-dimensional convolution-based ECNN-1D model for pear provenance identification. The model integrates an ECA module into a deep one-dimensional convolutional backbone to enhance its discriminative capability for Vis–NIR and SWIR spectral features. Experimental results demonstrated that ECNN-1D significantly outperformed traditional machine learning methods (LDA, RF, KNN, and SVM) as well as standard deep learning architectures (ResNet-1D, VGG-1D, and CNN-1D) across all test sets. This performance advantage can be primarily attributed to the ECA module, which dynamically selects and recalibrates critical channel features, allowing the model to focus on the most discriminative spectral information and more effectively distinguish pears from different origins. Notably, ablation experiments indicated that removing the ECA module, resulting in a standard CNN-1D architecture, still maintained relatively high classification performance but was clearly inferior to ECNN-1D in capturing subtle spectral differences across multiple spectral regions. These results indicate that the performance improvement primarily stems from the enhancement of key spectral features by the ECA module, rather than from an increase in network depth.

### 4.2. Comparison with Previous Studies

Compared with previous studies, the ECNN-1D model demonstrated superior performance in the geographical origin classification of fragrant pears. Early studies using hyperspectral imaging combined with support vector machines achieved an accuracy of 91.67%, indicating limited capability of traditional machine learning methods in modeling complex nonlinear features in high-dimensional spectral data [37]. Subsequently, hyperspectral imaging combined with two-dimensional convolutional neural networks improved classification accuracy to 97.7%, but relied primarily on spatial features and underutilized spectral sequence information [38]. A customized convolutional neural network (CNN-S) based on 450–1000 nm hyperspectral data achieved 93.5% accuracy for Korla fragrant pear maturity classification; however, its ability to distinguish subtle inter-origin spectral differences was not validated [39]. Recent studies integrating hyperspectral imaging with intelligent optimization algorithms have improved model stability and classification performance to a certain extent. However, these approaches primarily rely on parameter optimization and traditional feature engineering, which limits their ability to automatically learn deep spectral feature representations [40]. In contrast, the proposed ECNN-1D model incorporates an Efficient Channel Attention mechanism into a one-dimensional convolutional architecture, enabling adaptive enhancement of key spectral channels and suppression of redundant information. This design effectively exploits spectral sequence features while maintaining low model complexity, resulting in improved feature representation and classification performance.

### 4.3. Limitations and Future Research Directions

Despite the high accuracy of the ECNN-1D model in fragrant pear provenance identification, several challenges remain for its commercialization. First, this study primarily relies on limited samples from a single harvest year, which may not fully capture the phenotypic variability across different harvest years, storage conditions, and cultivars, thereby restricting the model’s generalizability. Second, variations in illumination, fruit orientation, and other environmental factors in production settings can introduce noise and spectral drift in hyperspectral data, adversely affecting classification accuracy. Although dimensionality reduction methods such as PCA and UMAP can effectively reduce data dimensionality and improve computational efficiency, real-time processing of high-resolution Vis–NIR and SWIR data still imposes substantial demands on hardware and software, particularly in large-scale commercial inspection scenarios. Future studies should expand the sample coverage and validate multi-batch and multi-cultivar datasets to enhance the model’s robustness and generalizability while exploring deep integration with automated sorting systems to support efficient and reliable commercial provenance identification.

## 5. Conclusions

This study proposed an ECNN-1D for the origin identification of fragrant pears, which incorporates an ECA mechanism and utilizes hyperspectral imaging data for precise origin discrimination. By preprocessing and performing feature dimensionality reduction on Vis–NIR and SWIR spectral data, and systematically comparing ECNN-1D with traditional machine learning models (LDA, RF, KNN, SVM) and representative deep learning models (VGG-1D, ResNet-1D, CNN-1D), the results showed that ECNN-1D achieved a test set accuracy of 100.00% on the Vis–NIR spectra, and 98.94% on the SWIR spectra, with an F1 score of 98.95%, significantly outperforming other methods, demonstrating superior feature extraction capability, classification stability, and generalization potential. Ablation studies further validated the effectiveness of the channel attention mechanism in capturing informative spectral features and enhancing discriminative ability, indicating that the ECA module can significantly improve classification performance in certain network architectures and spectral conditions, particularly for the more challenging SWIR spectral data. Dimensionality reduction analysis demonstrated that PCA and UMAP effectively preserved critical spectral information, with the Vis–NIR spectra showing clear class separation in the feature space, further supporting the model’s ability to fully leverage discriminative spectral bands.

In summary, this study demonstrates that the ECA-based ECNN-1D model can achieve high-precision, non-destructive fragrant pear origin identification, providing a reliable tool for fruit traceability, quality supervision, and standardized testing. Future work will focus on expanding field samples across multiple growing regions, harvest years, and storage conditions to construct a more diverse and representative dataset, thereby enhancing the model’s robustness and generalization under varying environmental conditions, further validating its performance on large-scale commercial batches and different fragrant pear cultivars, and to provide technical support for commercial applications and hyperspectral detection of similar agricultural products.

## Figures and Tables

**Table 1:** Sample distribution of pear varieties across different storage periods.

Storage Period	Days	Description	Hongxiangsu Pear	Aksu Pear	Korla Pear	Total
Initial	1	Fresh harvest	60	60	60	180
Short-term	6	High freshness	60	60	60	180
Mid-term	11	Gradual change	60	60	60	180
Long-term	16	Deterioration	60	60	60	180
Total	—	—	240	240	240	720

**Table 2:** Parameters of Hyperspectral Imaging Camera.

Parameter	SOC710-SWIR	SOC710-VP
Spectral range	915–1700 nm	375–1045 nm
Spectral resolution	2.72 nm	4.09 nm
Number of spectral bands	288	128
Sensor material	InGaAs	Silicon
Spatial resolution	640×512 pixels	696×512 pixels
Pixel pitch	25 μm	4.65 μm
Lens model	Kowa LM50HC	Schneider CITRINE
Aperture range	F1.4–F16	F1.4–F11
Fixed focal length	50.0 mm	17.0 mm

**Table 3:** Training settings of the deep learning models.

Item	Value
Batch size	8
Initial learning rate	5 × 10^−4^
Seeds	5
Weight decay	1 × 10^−5^
Optimizer	AdamW
Patience	10
Epochs	50

**Table 4:** Classification performance of different models based on Vis–NIR and SWIR hyperspectral data. Note: ^†^ = best, * = worst.

Spectra	Model	Validation (%)	Test (%)	P (%)	R (%)	F1 (%)
Vis–NIR	LDA	100.00 ± 0.00	100.00 ± 0.00	100.00 ± 0.00	100.00 ± 0.00	100.00 ± 0.00
	RF	99.39 ± 0.57	99.85 ± 0.30	99.86 ± 0.32	99.84 ± 0.35	99.85 ± 0.34
	KNN	99.70 ± 0.37	99.55 ± 0.37	99.56 ± 0.41	99.53 ± 0.43	99.54 ± 0.42
	SVM	100.00 ± 0.00	100.00 ± 0.00	100.00 ± 0.00	100.00 ± 0.00	100.00 ± 0.00
	VGG-1D	100.00 ± 0.00	100.00 ± 0.00	100.00 ± 0.00	100.00 ± 0.00	100.00 ± 0.00
	ResNet-1D	100.00 ± 0.00	99.55 ± 0.61	99.57 ± 0.64	99.52 ± 0.71	99.54 ± 0.69
	CNN-1D	100.00 ± 0.00	99.70 ± 0.61	99.72 ± 0.63	99.68 ± 0.71	99.69 ± 0.69
	ECNN-1D	100.00 ± 0.00	100.00 ± 0.00	100.00 ± 0.00	100.00 ± 0.00	100.00 ± 0.00
SWIR	LDA	97.40 ± 0.61	97.73 ± 0.68	97.74 ± 0.74	97.78 ± 0.74	97.73 ± 0.77
	RF	97.25 ± 1.42	95.91 ± 0.91	95.98 ± 0.98	95.88 ± 1.02	95.90 ± 1.01
	KNN	96.21 ± 1.66	96.36 ± 1.30	96.42 ± 1.39	96.35 ± 1.44	96.35 ± 1.44
	SVM *	96.06 ± 1.47	95.76 ± 1.56	95.84 ± 1.65	95.71 ± 1.74	95.74 ± 1.71
	VGG-1D	98.94 ± 0.68	97.88 ± 0.88	97.91 ± 0.94	97.87 ± 1.01	97.87 ± 0.98
	ResNet-1D	97.42 ± 2.56	95.91 ± 2.17	96.02 ± 2.40	95.95 ± 2.41	95.90 ± 2.45
	CNN-1D	98.94 ± 0.61	98.03 ± 1.23	98.20 ± 1.18	97.99 ± 1.43	98.03 ± 1.37
	ECNN-1D ^†^	99.09 ± 0.74	98.94 ± 0.77	98.96 ± 0.74	98.96 ± 0.76	98.95 ± 0.76

**Table 5:** Ablation study results of the ECA module on different deep learning models.

Spectra	Model	Validation (%)	Test (%)
Vis–NIR	VGG-1D	100.00 ± 0.00	100.00 ± 0.00
	VGG-1D+ECA	100.00 ± 0.00	100.00 ± 0.00
	ResNet-1D	100.00 ± 0.00	99.55 ± 0.61
	ResNet-1D+ECA	100.00 ± 0.00	99.39 ± 0.63
	CNN-1D	100.00 ± 0.00	99.70 ± 0.61
	ECNN-1D	100.00 ± 0.00	100.00 ± 0.00
SWIR	VGG-1D	98.94 ± 0.68	97.88 ± 0.88
	VGG-1D+ECA	98.64 ± 1.01	98.33 ± 0.88
	ResNet-1D	97.42 ± 2.56	95.91 ± 2.17
	ResNet-1D+ECA	98.79 ± 0.41	95.76 ± 1.74
	CNN-1D	98.94 ± 0.61	98.03 ± 1.23
	ECNN-1D	99.09 ± 0.74	98.94 ± 0.77

**Table 6:** Accuracy of single-source fragrant pear origin identification models based on PCA-reduced features. Note: ^†^ = best, * = worst.

Spectra	Model	Validation (%)	Test (%)
Vis–NIR	LDA	100.00 ± 0.00	100.00 ± 0.00
	RF	99.85 ± 0.30	99.55 ± 0.61
	KNN	99.70 ± 0.37	99.55 ± 0.37
	SVM	100.00 ± 0.00	100.00 ± 0.00
	VGG-1D	100.00 ± 0.00	100.00 ± 0.00
	ResNet-1D	100.00 ± 0.00	99.55 ± 0.61
	CNN-1D	100.00 ± 0.00	99.55 ± 0.61
	ECNN-1D	100.00 ± 0.00	99.70 ± 0.37
SWIR	LDA	98.64 ± 0.74	97.58 ± 1.47
	RF	98.03 ± 0.77	97.58 ± 1.30
	KNN	96.21 ± 1.66	96.36 ± 1.30
	SVM *	96.21 ± 1.27	95.91 ± 1.63
	VGG-1D	98.48 ± 0.68	97.73 ± 1.27
	ResNet-1D	98.18 ± 0.91	96.97 ± 1.59
	CNN-1D ^†^	98.33 ± 0.88	97.88 ± 0.88
	ECNN-1D	98.18 ± 0.91	96.82 ± 1.94

**Table 7:** Accuracy of single-source fragrant pear origin identification models based on UMAP-reduced features. Note: ^†^ = best, * = worst.

Spectra	Model	Validation (%)	Test (%)
Vis–NIR	LDA	99.24 ± 0.83	98.33 ± 0.88
	RF	99.09 ± 0.57	99.24 ± 0.68
	KNN	99.24 ± 0.48	99.55 ± 0.37
	SVM	98.64 ± 0.88	99.09 ± 0.57
	VGG-1D	99.09 ± 0.57	98.94 ± 0.77
	ResNet-1D	99.24 ± 0.48	99.24 ± 0.68
	CNN-1D	99.24 ± 0.48	99.09 ± 0.88
	ECNN-1D	98.94 ± 0.61	98.79 ± 0.77
SWIR	LDA	92.73 ± 2.12	91.97 ± 2.28
	RF	93.79 ± 3.08	92.42 ± 2.49
	KNN	94.24 ± 2.17	93.33 ± 1.30
	SVM *	87.58 ± 7.43	88.33 ± 4.19
	VGG-1D	93.03 ± 2.97	92.12 ± 1.23
	ResNet-1D	95.45 ± 2.03	92.73 ± 2.23
	CNN-1D	95.30 ± 2.22	93.94 ± 2.67
	ECNN-1D ^†^	95.61 ± 1.30	94.24 ± 2.17

## Data Availability

The original contributions presented in the study are included in the article; further inquiries can be directed to the corresponding authors.
